# Supplementary material for: Stacking Model‐Based Classifiers for Dealing With Multiple Sets of Noisy Labels
Source: Biom J. 2025 Mar 12;67(2):e70042. doi: 10.1002/bimj.70042 (PMC11898607; doi:10.1002/bimj.70042)
Supplement: Supplementary file 1 — Supporting Information [file BIMJ-67-e70042-s001.zip › density-based-ensemble-model-main/competitors_real_data/netlab-master/netlab-master/help/demprgp.htm]

Netlab Reference Manual demprgp


# demprgp

## Purpose

Demonstrate sampling from a Gaussian Process prior.

## Synopsis

```
demprgp
```

## Description

This function plots the functions represented by a Gaussian Process
model. The hyperparameter values can be adjusted
on a linear scale using the sliders (though the exponential
of the parameters is used in the covariance function), or
by typing values into the text boxes and pressing the return key.
Both types of covariance function are supported. An extra function
specific parameter is needed for the rational quadratic function.

## See Also

`gp`

---

**Pages:**
Index


---

Copyright (c) Ian T Nabney (1996-9)
